# Perceived Risk for Dengue Infection Mediates the Relationship between Attitude and Practice for Dengue Prevention: A Study in Seremban, Malaysia

**DOI:** 10.3390/ijerph192013252

**Published:** 2022-10-14

**Authors:** Mohd ‘Ammar Ihsan Ahmad Zamzuri, Farah Nabila Abd Majid, Rahmat Dapari, Mohd Rohaizat Hassan, Abd Majid Mohd Isa

**Affiliations:** 1 Department of Community Health, Faculty of Medicine, National University of Malaysia Jalan Yaacob Latif, Bandar Tun Razak, Cheras, Kuala Lumpur 56000, Malaysia; 2 Seremban District Health Office, Ministry of Health Malaysia, Jalan Lee Sam, Bandar Seremban, Seremban 70590, Malaysia; 3 Malaysian Society for Environmental Epidemiology (MySEE), Persiaran Taman Melati, Taman Melati, Setapak, Kuala Lumpur 53100, Malaysia; 4 Department of Psychiatry, Hospital Canselor Tuanku Muhriz, Faculty of Medicine, National University of Malaysia Jalan Yaacob Latif, Bandar Tun Razak, Cheras, Kuala Lumpur 56000, Malaysia; 5 Department of Community Health, Faculty of Medicine and Health Science, Universiti Putra Malaysia (UPM), Serdang 43400, Malaysia; 6 Borneo Medical and Health Research Centre, Faculty of Medicine and Health Sciences, University Malaysia Sabah, Kota Kinabalu 88400, Malaysia; 7 Faculty of Education and Liberal Arts, INTI International University, Persiaran Perdana BBN Putra Nilai, Nilai 71800, Malaysia

**Keywords:** dengue, risk perception, mediator, attitude, practice, structural equation modelling

## Abstract

Dengue remains a public health concern due to limited curative treatment and safe vaccine availability. Prevention by individual is utmost necessary but its practice is still lacking. Perceived risk to disease has been reported to exert a good effect on health behaviour change. However, limited evidence showed its relationship towards attitude and practice for dengue prevention. Hence, we aim to measure the mediating effect of dengue risk perception in the relationship between dengue attitude and dengue prevention practice. A cross-sectional study was conducted from April 2021 to November 2021 in a district of Seremban using a pre-validated questionnaire. Informed consent was obtained from the respondents prior to inclusion in the study. The study was approved by the ethical research committee. A total of 347 respondents took part in the survey, but only 341 data points were included in the final analysis. The majority of the respondents were female (63.0%), of Malay ethnicity (86.8%), married (55.4%), and currently employed (71.0%). The pooled confirmatory factor analysis result demonstrated an RMSEA value of 0.038 (<0.08), CFI value of 0.969 (>0.90), TLI value of 0.9565 (>0.90), and *ChiSq/df* = 1.479 (<3.0). All the hypotheses for direct effect yielded a significant and positive relationship. Bootstrapping analysis to test for mediation revealed a partial mediation effect as both indirect and direct effects are significant. Risk perception is a mediator variable between attitude and dengue prevention practice. Therefore, our recommendation is to increase health awareness activity that helps to improve individual’s risk perception through active health promotion and a health educational campaign that inculcates dengue risk messages. Ultimately, this effort can enhance good health prevention behaviour.

## 1. Introduction

Dengue fever is the most important vector-borne viral disease in the world, affecting 129 countries. The World Health Organisation (WHO) estimates that nearly 3.9 billion people are at risk of dengue fever infection and gives projections of 96 million symptomatic cases and 40,000 deaths every year globally [1]. The South East Asia (SEA) region is responsible for more than half of the global dengue fever burden, and five countries in this region (India, Indonesia, Myanmar, Sri Lanka, and Thailand) are present in the top 30 countries with high dengue endemicity [2]. Data reported by WHO demonstrated an increase in the number of dengue fever cases in the SEA region from 451,442 to 658,301 cases from 2015 to 2019, respectively. This reported increase highlights the urgency of controlling and, ultimately, ending this endemic disease.

Affected countries are still struggling to address dengue due to the multifaceted aetiology of dengue transmission, the absence of curative treatments, and the fact that a safe and efficacious dengue vaccine is unavailable. As a result, strategies that aim to combat pervasive incidences of dengue fever significantly depend on vector control and continuous surveillance programmes [3]. Specifically, traditional vector control modalities rely on top-down approaches (i.e., authority to community) to reduce transmission through physical (destruction or manipulation of water-holding containers), biological (use of bacteria or guppy fish), and chemical means (use of larvicides and space spraying) [4]. 

Although these vector control efforts have had some success, they can only provide control in the short term, as regions using these strategies have previously been re-infested within a short time. Therefore, a paradigm shift towards the use of bottom-up approaches has occurred to strengthen participatory involvement at the community level to address this issue. For example, one of the strategies recommended by WHO involves promoting significant behavioural changes through effective communication and social mobilisation, known as COMBI [5]. The platform endorses public awareness activities that support communities by disseminating key messages about the disease and equipping them with the ability to prevent dengue transmission at the community level. Through this, the sustainability of vector control activities in the long term can be ensured, as communities adopt appropriate preventative behaviours against dengue fever. 

Adapting to new behaviour patterns after many years of living comfortably with particular lifestyle is a challenging process, but it is achievable. Indeed, according to Health Belief Model (HMB) theory, healthy behaviour changes can occur in response to perceived health risks, which are the personal evaluations of hazards to which the individual is or may be exposed [6]. Therefore, the reaction of the individual is dependent on their subjective interpretations of the risk. For instance, individuals may adopt good health practices if there is a high perceived risk of getting the disease, and vice versa [7]. Overall, the HBM model illustrates the diverse constructs that influence behaviour changes, including perceived severity of the illness, perceived barriers, perceived benefits, cues to action, and self-efficacy [8]. 

**Figure 1:**
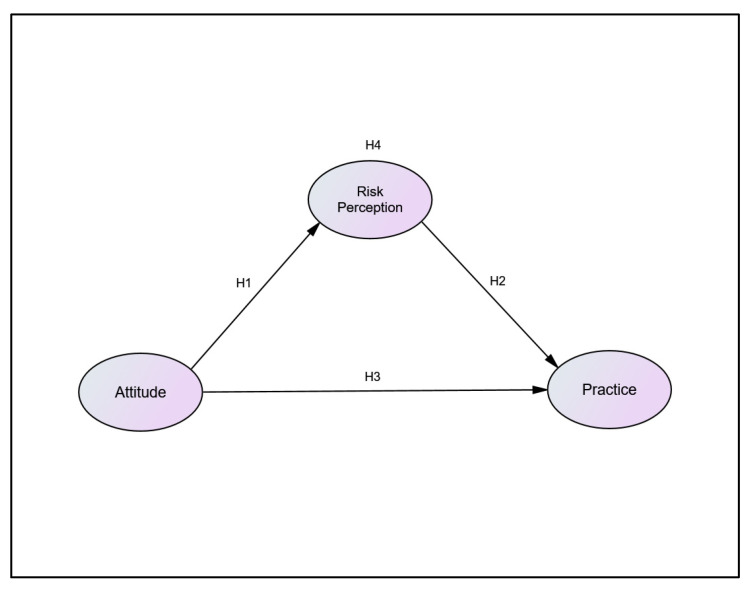
Theoretical framework of risk perceptions, attitudes, and dengue preventative practices.

However, previous literature focusing on dengue risk perceptions and dengue prevention practices is scarce, especially in terms of the complex interaction between these two factors and the construct of attitude. This paper proposes an interaction model among these three constructs, as shown in Figure 1, based on the theoretical framework developed by Lusk and Coble 2005 [9]. Furthermore, previous studies conducted by Walter and Emery 2006 and Pan et al., 2020 have found significant mediating effects of risk perception on human behaviour [10,11]. Therefore, analysing dengue-related risk perceptions that mediates preventative behaviours against dengue may significantly facilitate our understanding of the relationship between these factors, which can ultimately be utilised to enhance current COMBI activities.

Exploring the mediating effects of risk perception requires sophisticated statistical analysis techniques. Indeed, structural equation modelling (SEM) is capable of estimating the relationships within an integrated system and measuring the latent variables that cannot be directly observed [12]. To date, the application of SEM has been extended to a variety of areas, including the health field [13]. Specifically, two methods of SEM, covariance-based SEM (CB-SEM) and partial-least square SEM (PLS-SEM), have specific advantages and can be conducted using software for analysis, thus minimising errors and enhancing time efficiency. 

Based on the significant increasing trend of dengue incidence rates in Malaysia from only 72 cases per 100,000 population in 2001 to 361 cases per 100,000 population by 2014 [14], this study aims to explore the relationships between dengue risk perception, attitudes, and preventative practices. There are four main hypotheses in this study as shown in Table 1. We hypothesise that risk perception acts as a mediating variable that produces a positive effect towards dengue preventative practices. The findings of the study will be useful for developing targeted health messages for behavioural change in relation to dengue fever.

## 2. Materials and Methods

### 2.1. Study Design and Setting

This cross-sectional study was conducted between April 2021 and July 2021. During the respondent recruitment process, Malaysia began to implement a third lockdown with national movement control orders due to the increase in coronavirus disease 2019 (COVID-19) cases. Therefore, respondents were recruited in areas where groups of individuals were allowed to gather, such as at healthcare facilities (vaccination centres) and large hypermarkets (only for pilot testing but not reported in this study). Nevertheless, the study was conducted in adherence to strict social distancing policies and minimal physical contact was maintained as far as possible.

Malaysia is located in the region of south-east Asia and has been continuously burdened with dengue infection [14] as well as significant economic impact [15]. The geographical area of this study covered the district of Seremban in Malaysia. Seremban is the capital city of the Negeri Sembilan state and is located next to Selangor and Wilayah Persekutuan Kuala Lumpur. This area is also known as “Greater Klang Valley”. The total population of this district is estimated at approximately 482,512 people, of which approximately 77.2% (372,917 people) live in the major towns and cities [16]. As a result of its rapid development, the Seremban district accounted for nearly 90% of the dengue cases in the state of Negeri Sembilan. This figure is in line with the pattern in the capital cities of other districts that also suffer from a high burden of dengue fever, such as Petaling district, Hulu Langat district, and Johor Bharu district. Therefore, the similarity of Seremban to other cities ensures the external validity of the results.

### 2.2. Data Collection

**Figure 2:**
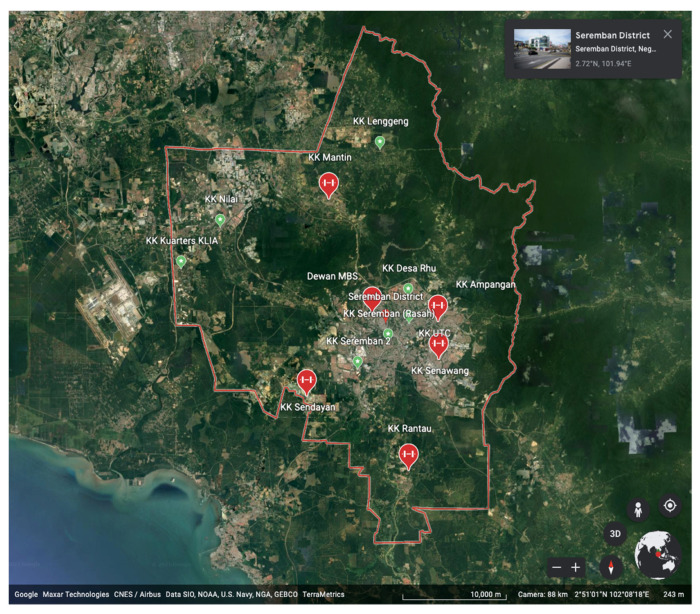
Mapping of study location based on cluster of health facilities.

The respondents in this study were recruited using a multistage sampling technique. To obtain as representative a sample as possible, the survey was conducted at 6 locations randomly selected from 20 available options, Figure 2. These 20 health facilities were clustered together according to their geographical proximity and administrative territories, to ensure optimisation of resources and feasibility of the study. These locations serve as COVID-19 vaccination centres that can hold more than 300 people at a given time. Since the name list is available 3 days earlier, we worked to the best of our ability to achieve maximum randomness by selecting the names prior to data collection time. However, due to unforeseen circumstances, some selected respondents did not make it into the study, and, thus, required substitution to ensure a large enough sample size to conduct the analysis with good power.

The number of respondents used was derived from the calculation of the lead author’s main study, which aims to predict factors affecting dengue risk perception [17]. Based on a formula by Lachenbruch et al. (1991) on determining sample size in health studies [18], and after computing the proportion of 0.71 [17], with a 5% margin of error, confidence level of 95%, and attrition rate of 10%, the number of required respondents in the sample is 347. Although the outcome of the main study was not presented here, this number is adequate to perform structural equation modelling, where a range between 300 and 500 samples is sufficient when analysing at least seven constructs and a minimum of three items per construct [19]. 

During the data collection process, the respondents were given the option to answer the survey using either a conventional survey form or an online Google Form, which was readily available to be scanned and downloaded. Only one response was counted for each household, and it was preferable for the main earner of the family to be recruited rather than other family members. Additionally, only respondents over 18 years of age who gave verbal consent were eligible to participate in the survey. Finally, no action was taken regarding those respondents who did not complete the survey, as survey participation was on a voluntary basis.

### 2.3. Study Instrument

The survey tool used to measure the mediating effects of the preventative practices against dengue was adapted from a validated questionnaire developed earlier by the researcher [20]. The Health Belief Model (HBM) theory is widely known, and it has evolved several times since its development. It is not used to measure risk perception per se, but its theoretical model has helped the researcher to build the domain in the questionnaire following meticulous discussion with the questionnaire development expert team [21] as well incorporation with the “Standard Questionnaire on Risk Perception of An Infectious Disease Outbreak” as references [22]. Nevertheless, the HBM constructs have been adopted in variety of ways to develop tools to measure risk perception [7,23,24,25]. Thus, we believe that the flexibility in the constructs serves as its advantages and versatility. 

There were four sections (A, B, C, and D) in the questionnaire. Firstly, the risk perception section was formulated based on HBM theory, and it examined the domains of perceived susceptibility to disease, perceived severity of the disease, perceived barriers, and perceived benefits. Secondly, the attitude domain was constructed from the HBM sub-domains of self-efficacy and cues to action. Thirdly, the preventative practices against dengue related to actions conducted both at home and outside the house. The scale is assumed to be of higher order with type 1 reflective–reflective constructs. It yielded good fit indices based on the confirmatory factor analysis upon its development, with a root mean square area of approximation (RMSEA) value of 0.061, a standardised root means square residual (SRMR) of 0.068, a parsimonious normed fit index (PNFI) of 0.649, and a goodness of fit index (GFI) of 0.996.

(a) Section A: demographic data.

Collect baseline characteristics of the respondents.

(b) Section B: risk perception (4 sub-domains)

There are 12 items with a 5-point Likert scale. Five items are negatively worded, thus requiring reverse scoring to obtain the final summation score for the risk perception construct. The mediation analysis used a continuous variable dataset that did not require any summation process.

(c) Section C: attitude

There are seven items with a 5-point Likert scale for this domain. All items are positively polar. The analysis for mediation did not require any item to be added up.

(d) Section D: practice

There are ten items with a 5-point Likert scale for this domain. All items are positively polar. The analysis for mediation did not require any item to be added up.

### 2.4. Data Analysis

Data collected from the survey form were entered into a Microsoft Excel 365 (Version 16.65) spreadsheet, and data from the online Google Form were downloaded before all the data were integrated. Any duplicates and missing variables were removed. There were no duplications found and six were incomplete survey forms, thus were all rejected. Categorical data were presented using frequencies and percentages, whereas the continuous categories were interpreted using the medians and interquartile ranges (IQR). A normality test was conducted with the continuous data using the Kolmogorov–Smirnov test prior to analysis. The CB-SEM method was employed for the mediation analysis by using IBM-SPSS-AMOS software. Two approaches were used to test the mediation effect complimentarily. Initially, the hypothesis was tested conventionally, which is comparing the result of direct effects and indirect effects through path analysis. Subsequently, the confirmation of the result was performed using the bootstrapping method. The study performed the bootstrapping procedure using 1000 samples with both percentile confidence intervals and a bias-corrected confidence interval of 95%. Results from these analyses yield the coefficients and *p*-values of the pathways. The significance value *p*-value was set at < 0.05 to be statistically significant.

### 2.5. Ethical Approval

This study was approved by Universiti Kebangsaan Malaysia (UKM) Research Ethics Committee **(UKM PPI/111/8/JEP-2019-854)** and registered with National Medical Research Register (NMRR). The study abides to the Helsinki declaration. Informed consent was obtained prior to participation in the study. There was a specific section in the paper form as well as in the Google Form to ensure consent was obtained before respondents could answer the survey. Apart from that, only respondents aged 18 years and above participated in this study. Ultimately, the data obtained was kept strictly confidential.

## 3. Results

A total of 347 respondents participated in this study, but only 341 data points were included in the final analysis. Table 2 demonstrates the baseline characteristics of the respondents in the analysis. The median age of respondents was 33 years old, with an IQR of 12. The majority of the respondents were female (63.5%, *N* = 215); married (55.4%, *N* = 189); currently employed (71.0%, *N* = 242); owned their house (73.9%, *N* = 252); had no personal dengue history (84.5%, *N* = 288) or family dengue history (56.3%, *N* = 192); and was not living in a dengue outbreak area (64.8%, *N* = 221). Table 3 shows the items and constructs of the questionnaire together with the normality test. 

**Figure 3:**
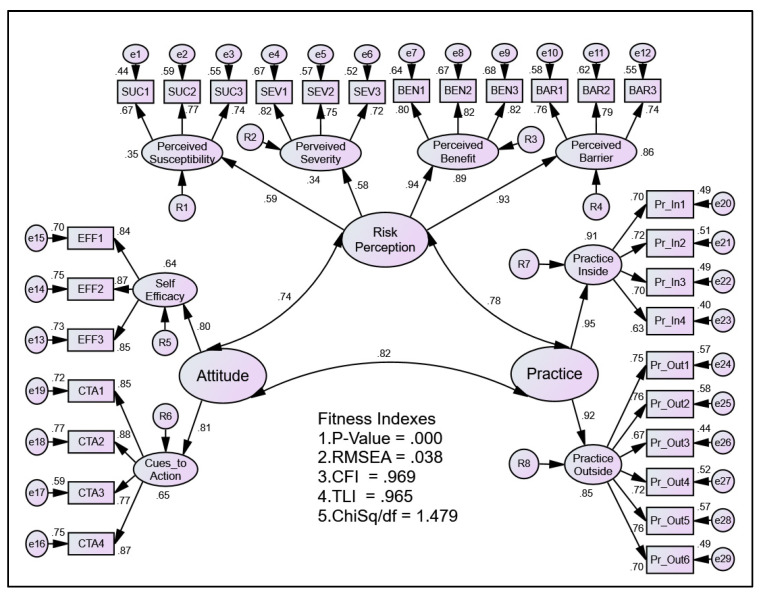
The results of Pooled-CFA show factor loading for all components as well as all items measuring their respective components.

The measurement model combining all latent constructs in the model is presented in Figure 3. The figure also depicts the validation of all the constructs together at once using the pooled Confirmatory Factor Analysis (CFA). The measurement model achieved a good model fit with an RMSEA value of 0.038 (<0.08), CFI value of 0.969 (>0.90), TLI value of 0.965 (>0.90), and ChiSq/df of 1.479 (<0.50). The CFA also revealed construct validity, convergent validity, discriminant validity, and composite reliability of these constructs, as shown in Table 4 and Table 5.

**Figure 4:**
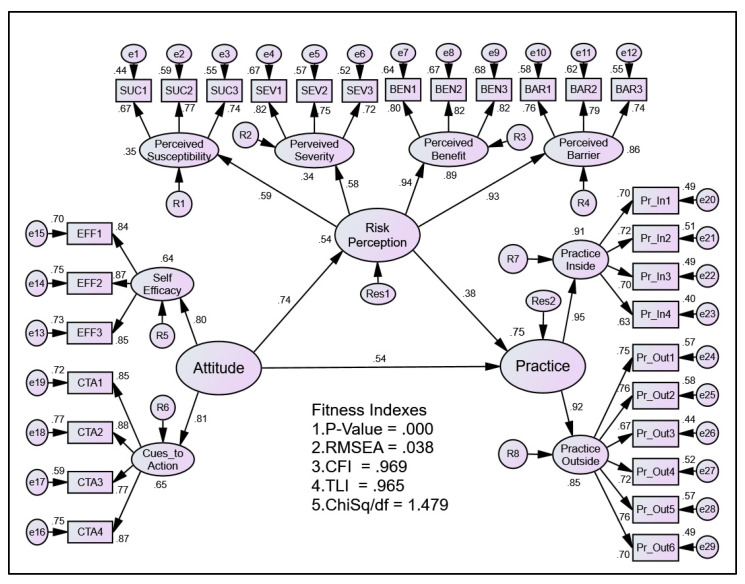
The results for standardised regression coefficient between constructs.

**Figure 5:**
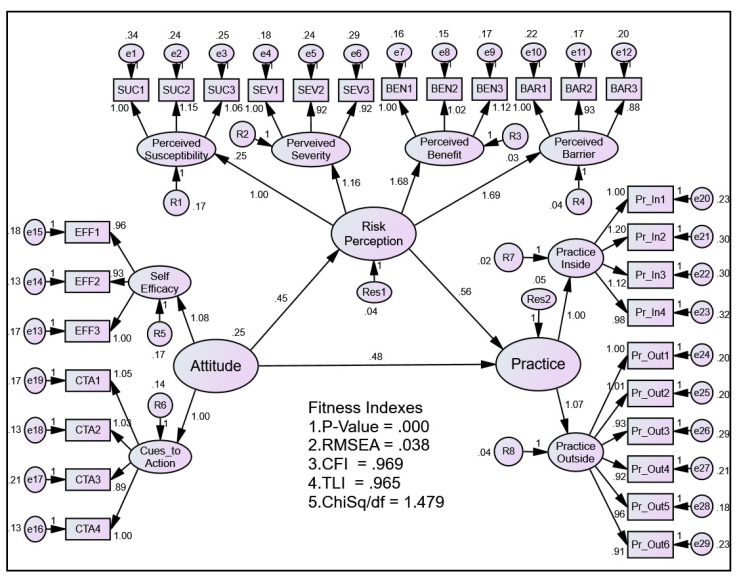
The results for regression coefficient between constructs.

Once the CFA procedure was completed and all constructs had passed the requirement for validity, reliability, and normality, the structural model and estimate of the parameter through the procedure of structural equation modelling (SEM) was performed. The structural model showed the domain attitude, as perceived by the respondents, contributed about 54 percent of their risk perception, while both attitude and risk perception domains, as perceived by the respondents, contributed about 75 percent of their dengue practice, Figure 4 (standardised regression). Nevertheless, when the attitude domain increased by one standard deviation, the risk perception domain increased by 0.45 standard deviation. On the other hand, when the attitude domain increased by one standard deviation, the practice domain increased by 0.48 standard deviation. Apart from that, when the risk perception domain increased by one standard deviation, the practice domain increased by 0.56 standard deviation, Figure 5 (unstandardised regression).

**Figure 6:**
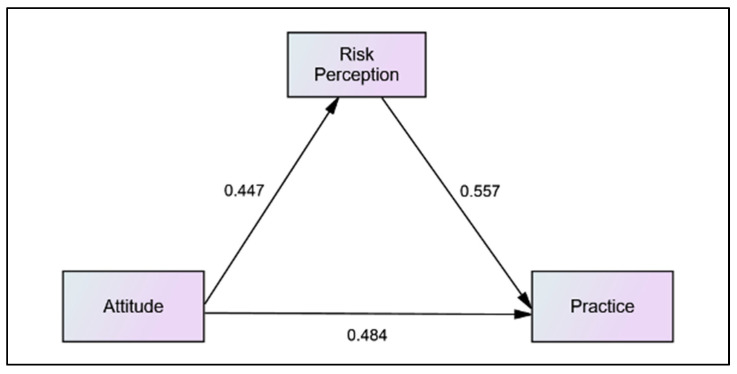
Path analysis of direct and indirect effect tested.

The result of mediation test using path analysis is shown in Figure 6. Both indirect effects between attitude and risk perception (0.447) and risk perception and practice (0.557) together with the direct effect between attitude and practice yielded a statistically significant result (*p* < 0.05). Thus, this demonstrated a partial mediation effect of these relationships. The subsequent bootstrap procedure confirmed the result, as shown in Table 6. The full result of the study hypothesis is shown in Table 7.

## 4. Discussion

The aim of this study was to determine the mediating effect of perceived risk of dengue infection on the relationship between dengue prevention attitudes and dengue prevention practices among the population of Seremban. The district of Seremban was selected due to the highest recorded number of dengue cases and dengue outbreak for the state of Negeri Sembilan [26]. Nevertheless, the higher rate of dengue infection has resulted a significant economic burden to the state’s healthcare cost in treating the spectrum of dengue presentation [27]. The economic burden faced by the state is similar to other ASEAN countries that suffer from dengue endemicity [28,29]. Hence, the need for conducting the study in Seremban is imperative to further the understanding of the issue.

The sample size used in the study is sufficient to allow the analysis for moderating effect by CB-SEM using IBM-SPSS-AMOS software. Traditionally, sample size used may vary between 100 to 200 [30] or by applying the rules-of-thumb, such as five or ten observations per estimated parameter [31]. As the country was facing a continuous lockdown episode for a long duration during the implementation of the study, our sample size could be valued as reasonable to ensure good power and generate a reliable result.

The characteristics of respondents in the study may not be a good representation of the district population. Taking age, for instance, in which the age distribution in this study was younger. This was inevitable as the older population was reserved from joining the study as they are categorised as a high-risk group from COVID-19 infection [32]. Furthermore, at the time of the study, the country had just recovered from the surge of COVID-19 mortality cases with a significant proportion of brought-in-death (BID) cases [33]. Considering the holistic view of the condition and also the remnant fear of the population, selection into the sampling frames was meticulously conducted, especially elderly with multiple comorbidities. 

The pooled-CFA analysis in this study revealed a significant fitness index, thus fulfilling the prerequisite condition for SEM analysis [19,34]. This is essential to show that the model built is in agreement with the theoretical construct of the knowledge. Traditionally, the determination of model fitness was conducted using the chi square test (χ^2^) [35]. However, some scholars dispute the single measurement to represent model fitness, thus leading to integration of several measurement tests [36,37]. Essentially, the use of absolute fitness index and relative fitness index were used and interpreted concurrently [38]. As such, our pooled-CFA has demonstrated sufficient fitness indices of the built model.

Our study has demonstrated a significant mediating effect of risk perception in the relationship between attitude and practices. Such finding has been shown in other behavioral studies [39]. For instance, in the Common Sense Model of Self-Regulation (CSM) behavioral model, it places a fundamental importance of risk perception to encourage good health behavior. Nevertheless, it specifies the positive linear relationship between perceived risk and the change in behavior [10]. 

Apart from that, some schools of thought believe that risk perception is the prerequisite for any significant behavior change. This is because the risk perception will indirectly influence motivation for health practice amendment [40]. Moreover, the behavior modification process will take place if one is perceived to be in high risk for the disease [41,42]. This theory is in line with a study conducted in Singapore that demonstrated a significant dengue preventive action (practice) in the community with high susceptibility to dengue transmission [43]. Likewise, the result concurred with the local study that has strong preponderance to the illness [44]. 

Nevertheless, a study using SEM analysis towards 497 respondents in the Republic of Guyana, Africa has shown a direct effect between perceived risk and health prevention practices [45]. It was found that with an increment of a unit score of perceived risk, it leads to an increment score of 0.53 units of preventive action. Thus, this depicts a direct relationship of these two constructs. Nonetheless, the application of perceived risk is not only limited in the case of dengue illness, as another study has demonstrated the direct effect of risk perception (harmful effect) of pesticides with the trend of its usage at a plantation [46].

### 4.1. Strengths and Limitations

This study has some key strengths. Firstly, this study utilised a validated tool that has been comprehensively analysed using dual statistical approaches, including Rasch measurement theory and confirmatory factor analysis. Additionally, the initial development of this tool for use with the local population ensured the tool was appropriate for the study setting. Secondly, the use of an SEM technique in the analysis gave power to the results [47]. Furthermore, the use of the parametric approach of CB-SEM, which is a second-generation method that is well established in confirmatory analyses, is also a major strength of this study [48]. Thirdly, the sample size for the study was both sound and sufficient, which is pertinent for ensuring the internal validity of the results. Finally, the integration of Google Form technology during the data collection saved time and enhanced cost-effectiveness. 

The main limitation of this study is the location of sample recruitment. As the respondents were recruited in places where gatherings were allowed, the data collected may have been biased towards certain types of respondents. For instance, the respondents recruited at the health centre may have good health-seeking behaviours, which could then be a potential confounder in this study. Secondly, the respondents may have incorrectly interpreted certain items on the self-administered questionnaire, thus leading to potential information bias. Thirdly, the ratio of the population sample that skewed towards a particular ethnicity could be the result of the bilingual tool used, which may negatively impact the external generalisation of the study result.

### 4.2. Recommendations

We recommend that the analytical technique of SEM should be widely used in medical and health-related studies due to the complex interplay of the factors under investigation. Indeed, using this method, the relationship of latent factors and constructs with a good theoretical framework can be assessed with specific populations and settings. Therefore, using this technique leads to a better understanding of the topic, which can be used to inform policymakers and clinicians, thus supporting them to make beneficial and impactful decisions. Despite its limitations, it is hoped that this study can become a reference for medical and health-related researchers to incorporate SEM in their study analyses.

## 5. Conclusions

The present study has demonstrated the mediating effect of perceived dengue risk in the relationship between attitude and dengue prevention practices. Therefore, the content of any health promotional activities and health education programmes should incorporate health messages that will stimulate the perceived threat of dengue infection such as element of severity, its complication, and possible recurrent outbreak in the neighbourhood. This specific and tailored information can help to enhance the population’s attitude and subsequently promote good dengue prevention practice. 

## Figures and Tables

**Table 1:** Hypotheses of the study and method of analysis.

	Hypothesis Statement	Statistical Analysis to Employ
H1	Attitudes have a positive effect on the Risk Perception among the population of Seremban.	Path Analysis in SEM
H2	Risk Perception has a positive effect on the Practice among the population of Seremban.	Path Analysis in SEM
H3	Attitudes have a positive effect on the Practice among the population of Seremban.	Path Analysis in SEM
H4	Risk Perception mediates the relationship between Attitudes and Practice among the population of Seremban.	Path Analysis in SEM and Bootstrapping

**Table 2:** Characteristics of respondents (*N* = 341).

No	Variable	*n* (%)
1.	Age *	33 (12)
2.	Gender	
	Male	126 (37.0)
	Female	215 (63.0)
3.	Ethnicity	
	Malay	292 (86.8)
	Chinese	17 (5.0)
	Indian	22 (6.5)
	Others	6 (1.8)
4.	Marital status	
	Single	152 (44.6)
	Married	189 (55.4)
5.	Employment status	
	Employed	242 (71.0)
	Unemployed	99 (29.0)
6.	House status	
	Owned	252 (73.9)
	Renting	89 (26.1)
7.	Personal dengue history	
	Yes	53 (15.5)
	No	288 (84.5)
8.	Family dengue history	
	Yes	149 (43.7)
	No	192 (56.3)
9.	Neighbourhood dengue outbreak	
	Current episode	120 (35.2)
	No dengue outbreak	221 (64.8)

* Median (interquartile range).

**Table 3:** Item scores.

Item	Domain	Code	Questions	Min	Max	Mean	Sd	Skew	c.r	Kurtosis	c.r
1	RP	SUC1	I am at risk to get dengue fever.	2.000	5.000	3.57	0.778	−0.077	−0.578	−0.197	−0.742
2	RP	SUC2	Dengue fever is a seasonal disease, I will be safe from it if the dengue season has passed.	2.000	5.000	3.43	0.770	−0.189	−1.422	0.039	0.147
3	RP	SUC3	I am bitten by mosquitoes every day, but I have never been infected with dengue fever. So, I am not at risk of getting dengue fever.	2.000	5.000	3.33	0.741	−0.029	−0.217	−0.225	−0.850
4	RP	SEV1	Dengue fever can cause death.	2.000	5.000	3.61	0.750	0.034	0.253	−0.282	−1.062
5	RP	SEV2	Fever for 3 days is worrisome to me. I feel that I cannot wait up to 5 days to get treatment.	2.000	5.000	3.44	0.752	−0.276	−2.078	−0.073	−0.274
6	RP	SEV3	I have many close friends who have recovered from dengue fever, but I am still afraid of dengue.	2.000	5.000	3.55	0.783	−0.061	−0.460	−0.240	−0.906
7	RP	BEN1	All the time and money I spent to stop dengue is worthwhile because I’m concerned about living a healthier lifestyle.	2.000	5.000	3.83	0.681	0.141	1.064	−0.495	−1.866
8	RP	BEN2	It is necessary for me to ensure there are no breeding spots around my house.	2.000	5.000	3.79	0.679	−0.065	−0.488	−0.442	−1.668
9	RP	BEN3	I need to be involved in every health campaign aimed to destroy mosquito breeding place, as it helps reduce the risk of dengue to my family.	1.000	5.000	3.77	0.739	−0.147	−1.106	−0.237	−0.892
10	RP	BAR1	I need a lot of money to implement dengue prevention at home.	2.000	5.000	3.82	0.734	−0.332	−2.502	0.208	0.786
11	RP	BAR2	I am very busy until I have no time to implement dengue prevention at home.	2.000	5.000	3.89	0.660	−0.341	−2.570	−0.190	−0.715
12	RP	BAR3	I need to spend the weekend with my family rather than participating in gotong royong to prevent dengue.	2.000	5.000	3.95	0.663	−0.439	−3.306	0.178	0.671
13	AT	EFF1	With at least one person who is knowledgeable about the disease in the house, he/she can help prevent the disease in the home.	2.000	5.000	3.94	0.778	−0.236	−1.778	−0.175	−0.660
14	AT	EFF2	It is necessary for me to deliver information about dengue fever to my family members.	2.000	5.000	3.90	0.733	−0.315	−2.376	0.006	0.021
15	AT	EFF3	I become more interested to take part in control/prevention of dengue when there is cooperation within the neighbourhoods.	1.000	5.000	3.99	0.799	−0.691	−5.206	1.040	3.922
16	AT	CTA1	It is necessary for me to ensure old and unused items that can store water, are kept closed.	1.000	5.000	3.83	0.777	−0.606	−4.572	1.037	3.908
17	AT	CTA2	It is necessary for me to ensure that the drainage or water flow system in my house to be properly maintained.	1.000	5.000	3.88	0.734	−0.806	−6.076	1.294	4.877
18	AT	CTA3	I only to dispose rubbish at the designated place.	2.000	5.000	3.88	0.721	−0.131	−0.989	−0.220	−0.830
19	AT	CTA4	I do not keep unused items that can store water.	2.000	5.000	3.84	0.722	−0.130	−0.977	−0.132	−0.499
20	PR	Pr-In1	I use mosquito repellent (lotion/spray/coil).	2.000	5.000	3.78	0.677	−0.098	−0.741	−0.411	−1.549
21	PR	Pr-In2	I always keep water containers in my house tightly closed.	2.000	5.000	3.53	0.791	−0.185	−1.395	−0.244	−0.920
22	PR	Pr-In3	I put larvicide into the water storage to kill the mosquito larvae.	2.000	5.000	3.73	0.762	−0.051	−0.388	−0.271	−1.021
23	PR	Pr-In4	I keep my drainage system properly maintained.	2.000	5.000	3.67	0.730	−0.397	−2.990	0.355	1.337
24	PR	Pr-Out1	I made complaint to the authority when I found an illegal dumping site.	1.000	5.000	3.72	0.690	−0.070	−0.525	−0.216	−0.813
25	PR	Pr-Out2	Abandoned and damaged vehicles in the neighbourhood trigger my intention to take the necessary action.	1.000	5.000	3.61	0.692	−0.429	−3.233	−0.031	−0.118
26	PR	Pr-Out3	I made complaint to the authority when there is damaged vehicle idling in my neighbourhood.	1.000	5.000	3.51	0.726	−0.399	−3.010	0.095	0.357
27	PR	Pr-Out4	I check for potential mosquito breeding place around the neighbourhood.	1.000	5.000	3.69	0.667	0.005	0.041	−0.155	−0.582
28	PR	Pr-Out5	I made complaint to the authority when I found illegal garden.	2.000	5.000	3.84	0.657	−0.097	−0.733	−0.430	−1.621
29	PR	Pr-Out6	I made complaint to the authority when I found illegal building structure.	1.000	5.000	3.76	0.674	−0.287	−2.160	0.073	0.275

Domain: RP = risk perception; AT = attitude; PR = practice Sd = standard deviation.

**Table 4:** The Average Variance Extracted (AVE) and Composite Reliability (CR).

Construct	Item	Factor Loading	CR (Above 0.6)	AVE (Above 0.45)
Attitude	Self-Efficacy	0.88	0.834	0.715
	Cues to Action	0.81		
Self-Efficacy	EFF1	0.84	0.889	0.728
	EFF2	0.87		
	EFF3	0.85		
Cues to Action	CTA1	0.85	0.893	0.697
	CTA2	0.88		
	CTA3	0.77		
	CTA4	0.87		
Risk Perception	P-Susceptibility	0.59	0.855	0.708
	P-Severity	0.58		
	P-Benefit	0.94		
	P-Barrier	0.93		
P-Susceptibility	SUC1	0.67	0.771	0.530
	SUC2	0.77		
	SUC3	0.74		
P-Severity	SEV1	0.82	0.808	0.584
	SEV2	0.75		
	SEV3	0.72		
P-Benefit	BEN1	0.80	0.854	0.662
	BEN2	0.82		
	BEN3	0.82		
P-Barrier	BAR1	0.76	0.807	0.583
	BAR2	0.79		
	BAR3	0.74		
Practice	P-Inside	0.95	0.933	0.874
	P-Outside	0.92		
P-Inside	Pr-In1	0.70	0.782	0.500
	Pr-In2	0.72		
	Pr-In3	0.70		
	Pr-In4	0.63		
P-Outside	Pr-Out1	0.75	0.873	0.534
	Pr-Out2	0.76		
	Pr-Out3	0.67		
	Pr-Out4	0.72		
	Pr-Out5	0.78		
	Pr-Out6	0.70		

**Table 5:** The discriminant validity index summary.

Construct	Attitude	Risk Perception	Practice
Attitude	0.85		
R-Perception	0.74	0.84	
Practice	0.82	0.78	0.93

**Table 6:** Result of bootstrapping analysis for mediator analysis.

	Indirect Effect (a × b)	Direct (c)
Bootstrapping Value	0.280	0.545
Probability Value	0.008	0.002
Results on Mediation	Significant	Significant
	Mediation exists since indirect effects are significant	
Type of Mediation	Partial Mediation since the direct effect is also significant	

**Table 7:** Final result of the study hypotheses.

	Hypothesis Statement	*p*-Value	Result
H1	Attitudes have a positive and significant effect on the Risk Perception among the population of Seremban.	0.001	Supported
H2	Risk Perception has a positive and significant effect on the Practice among the population of Seremban.	0.001	Supported
H3	Attitudes have a positive and significant effect on the Practice among the population of Seremban.	0.001	Supported
H4	Risk Perception mediates the relationship between Attitudes and Practice among the population of Seremban.	0.001	Supported

## Data Availability

All data are strictly confidential and reserved for the study. Please contact the authors for data requests.
